# An epidemiological and spatiotemporal analysis of visceral leishmaniasis in West Pokot, Kenya, between 2018 and 2022

**DOI:** 10.1186/s12879-024-10053-4

**Published:** 2024-10-16

**Authors:** Norbert J. van Dijk, Sherif Amer, Daniel Mwiti, Henk D. F. H. Schallig, Ellen-Wien Augustijn

**Affiliations:** 1 Department of Medical Microbiology and Infection Prevention, Laboratory for Experimental Parasitology, Amsterdam University Medical Centre, Meibergdreef 9, Amsterdam, 1105 AZ The Netherlands; 2 Amsterdam Institute for Immunology and Infectious Diseases, Infectious Diseases Programme, Amsterdam, The Netherlands; 3 Faculty of Geo-Information Science and Earth Observation (ITC), Department of Urban and Regional Planning and Geo-Information Management (PGM), University of Twente, Hallenweg 8, Enschede, 7522 NH The Netherlands; 4 Division of Vector-Borne and Neglected Tropical Diseases, Kenya Ministry of Health, Nairobi, Kenya; 5 Amsterdam Institute for Global Health and Development, Paasheuvelweg 25, Amsterdam, 1105 BP The Netherlands; 6 Faculty of Geo-Information Science and Earth Observation (ITC), Department of Geo-information Processing (GIP), University of Twente, Hallenweg 8, Enschede, 7522 NH The Netherlands

**Keywords:** *Leishmania donovani*, Visceral leishmaniasis, Spatiotemporal analysis, Kenya

## Abstract

**Background:**

Visceral leishmaniasis (VL) remains a significant public health concern in West Pokot County, Kenya, where a large outbreak between 2020 and 2022 emphasised the need for improved VL control strategies. However, these measures are partially hampered by limited insight into the geographical distribution of cases and localised outbreaks of the disease. This study aimed to describe the epidemiology and spatiotemporal patterns of VL in West Pokot between 2018 and 2022, in order to map the spread of VL transmission and identify regions that should be prioritised for control interventions.

**Methods:**

VL patient demographics and village of residence were retrieved from admission records of Kacheliba Sub-County Hospital in West Pokot, Kenya. The temporal trend in VL admissions between 2018 and 2022 was analysed using seasonal decomposition analysis. To describe the spatial distribution of VL cases, geographic coordinates of villages of residence were collected from pre-established databases, and VL incidence was mapped at the sub-location level. Hotspot analysis was performed per study year to identify villages with high VL incidence, and scan statistics were applied to detect spatiotemporal clusters of VL cases during the study period.

**Results:**

A total of 1948 VL patients were reported between 2018 and 2022. The annual number of cases increased from 245 in 2019 to 598 in 2022, and VL admissions were generally higher at the start of the wet seasons. 70% of the VL cases could be georeferenced, and mapping of VL incidence revealed high case rates in the east of West Pokot during the complete study period. The eastern villages Lotongot and Chepaywat were marked as VL hotspots at a 99% confidence level in all study years. In addition, five significant spatiotemporal clusters were detected in the east and north, suggestive of local VL outbreaks in these regions.

**Conclusions:**

The increase in VL hospital admissions during the study period stresses the need for enhanced VL control and outbreak mitigation in West Pokot. These control measures should be focused on the hotspot regions in the east of the county.

**Supplementary Information:**

The online version contains supplementary material available at 10.1186/s12879-024-10053-4.

## Background

Visceral leishmaniasis (VL), also known as kala-azar, is a severe neglected disease caused by the protozoan parasites *Leishmania donovani* and *Leishmania infantum*. These parasites are transmitted by phlebotomine female sandflies that feed on animals and humans for a blood meal. Clinical disease is characterised by chronic fever, splenomegaly and weight loss, and the fatality rate may be up to 95% when VL is left untreated [1]. Globally, there are an estimated 50,000 to 90,000 new VL cases every year [1]. These are mainly reported in East Africa and the Indian subcontinent, where VL is caused by anthroponotic *L. donovani*, and Brazil and the Mediterranean basin, which are endemic for zoonotic *L. infantum*.

Currently, Kenya has one of the highest VL incidence rates in the world [2]. Around 6 million people are considered to be at risk of infection, and official VL case reports increased from 950 new cases in 2017 to 1564 cases in 2022 [2, 3]. Several remote arid and semi-arid regions face endemic transmission of *L. donovani*, the only VL-causing *Leishmania* species in Kenya. One of the major VL foci is West Pokot County, located in the northwest of the country at the border with Uganda [4]. During a recent outbreak of VL across various parts of Kenya, West Pokot was most heavily affected, and 930 cases were officially reported between February 2020 and October 2022 [5].

In response to the nationwide increase in VL numbers, the Kenyan Ministry of Health (MoH) launched the first National Strategic Plan for Control of Leishmaniasis in 2021 [4]. Among its main objectives are the establishment and strengthening of surveillance systems for VL, monitoring of trends in epidemiology, and mapping of transmission foci. Currently, VL surveillance in Kenya relies on monthly reporting of diagnosed cases by VL treatment facilities [4]. These numbers are reported into the Kenya Health Information System (KHIS), which is accessible at the national level. As the number of health facilities with access to VL diagnostic tests and trained personnel remains limited, fine-scale data on the geographical spread of VL is currently unavailable. This complicates prioritisation of control measures and monitoring of disease expansion to previously unaffected regions [4].

In addition to VL surveillance by the Kenyan MoH, scientific research may provide insight into the spatial distribution of VL cases as well. However, only a handful of studies have mapped VL in Kenya below county level. These include a study by Ryan et al., which analysed spatial clustering of *Leishmania* seroprevalence in two villages in Baringo County [6], and another by Kanyina, which reported villages with the highest VL case load during an outbreak in Marsabit County in 2014 [7]. The only low-level spatial data from West Pokot were reported by *Médecins Sans Frontières* (MSF), who mapped the spatial distribution of VL at the village level between 2000 and 2010 [8]. In this period, most VL cases admitted to treatment centres originated from the southwest and north of the county. However, it is plausible that VL epidemiology in West Pokot has since changed, due to factors like population growth, the adoption of sodium stibogluconate with paromomycin (SSG-PM) as new primary VL treatment in 2012, and climate change affecting ecological aspects of the VL sandfly vector [9–14]. Additionally, this earlier study did not correct case numbers for differences in village population, nor did it statistically analyse spatial and temporal patterns of VL cases. This means that it is unclear which villages in West Pokot currently have the highest VL incidence, hampering efficient use of the limited resources available for VL control. Furthermore, elucidation of spatiotemporal dynamics of VL incidence, including possible seasonality in transmission, is warranted to improve outbreak preparedness and mitigation. To address these knowledge gaps, this study was performed to describe the epidemiology and spatiotemporal patterns of VL in West Pokot between 2018 and 2022, based on patient data from hospital records. More specifically, the objectives were to (1) describe the age and sex distribution of admitted VL patients; (2) describe the general temporal trend and investigate potential seasonality in VL hospital admissions during the study period; (3) visualise the spatial distribution of VL cases in West Pokot by mapping VL incidence at the sub-location level; (4) identify VL hotspots at the village level; (5) detect the occurrence of local spatiotemporal clusters (outbreaks) of VL during the study period. The outcomes of this study may guide targeting of VL control strategies at locations, populations and seasons where they are most needed. Moreover, they may highlight areas where provision of diagnosis and treatment services is required to improve access to VL care. Lastly, retrospective detection of spatiotemporal VL clusters will provide a basis for further research into possible predictors and drivers of these disease outbreaks.

## Methods

### Ethics statement

This study obtained ethical approval from the Amref Health Africa Ethics and Scientific Review Committee (ref. ESRC P1196/2022) and the National Association for Science, Technology and Innovation (ref. NACOSTI/P/22/18832) in Kenya. The study was approved by the Health Director of West Pokot County before initiation.

## Study area

**Fig. 1:**
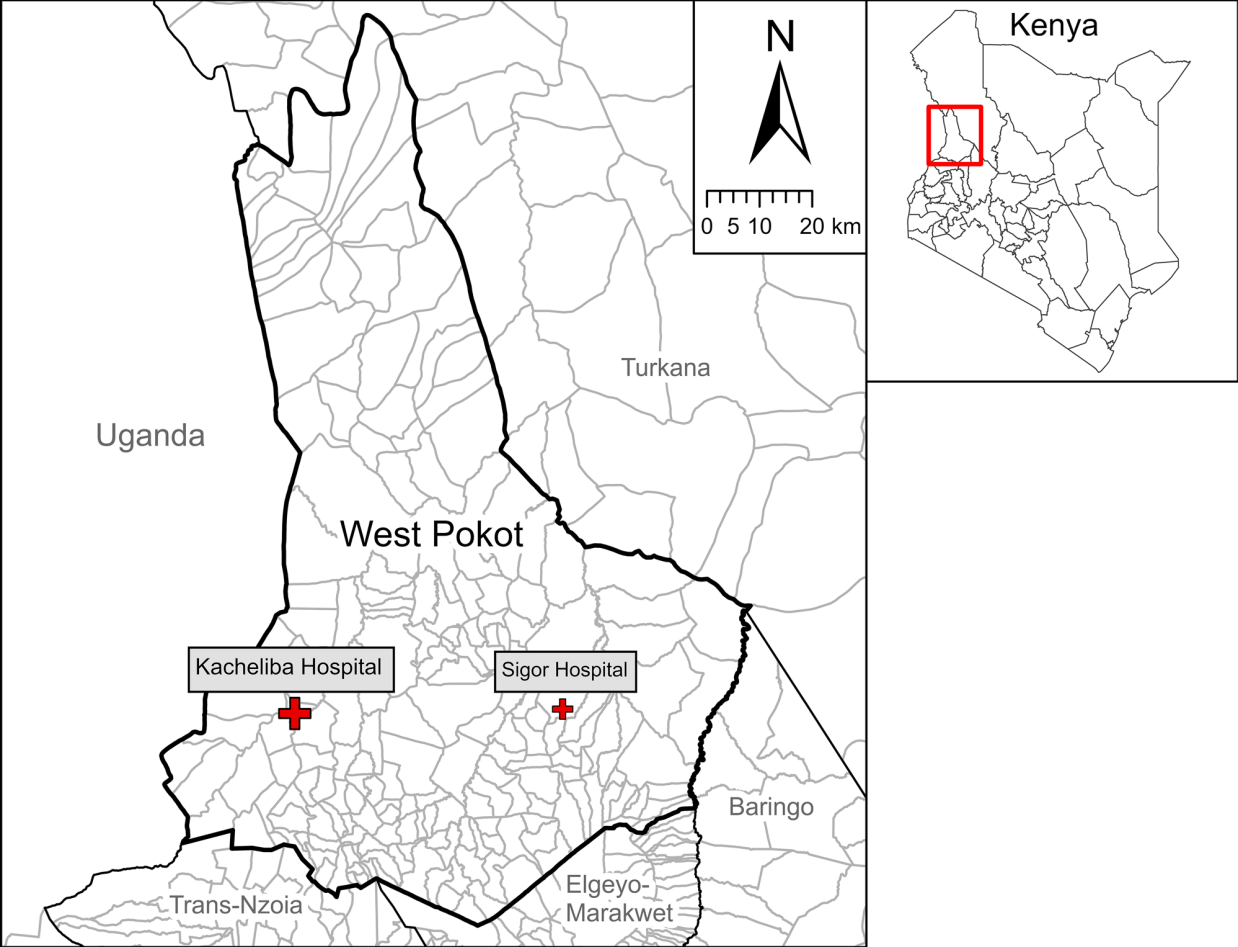
Map of West Pokot County and neighbouring counties with sub-locations. The study site at Kacheliba Sub-County Hospital has been highlighted, together with Sigor Sub-County Hospital as second centre for VL diagnosis and treatment in West Pokot

West Pokot County is located in the northwest of Kenya between 1.118°N – 2.671°N latitude and 34.784°E – 35.791°E longitude (Fig. 1). It covers an area of 9123 km^2^ and has an estimated population of 621,000 inhabitants [11]. Administratively, the county is divided into 4 sub-counties, 20 wards, 65 locations and 224 sub-locations. West Pokot has a semi-arid climate, with long rains from March to June and short rains from October to December. Approximately 95% of the population lives in remote villages, which are often poorly accessible due to the lack of tarmac roads and limited transport options [15]. These villages typically consist of scattered *manyattas*: compounds of a number of households with fencing of thorny *Acacia* branches. Most inhabitants of West Pokot belong to the Pokot tribe and traditionally have a pastoralist lifestyle.

West Pokot is highly endemic for VL, which is transmitted by female *Phlebotomus martini* sandflies. These insects mainly breed in termite mounds and are most active between dusk and dawn [16, 17]. There are four hospitals in West Pokot, of which two are currently offering VL diagnosis and treatment (Fig. 1): Kacheliba Sub-County Hospital (Suam location, Pokot North sub-county), which has been providing this service since 2006 and is the county’s reference centre for VL care; and Sigor Sub-County Hospital (Korellach location, Pokot Central sub-county), where a VL treatment centre was opened in September 2022.

## Data collection

Data were collected in November and December 2022 from handwritten paper records of the VL ward at Kacheliba Sub-County Hospital. Although Sigor Sub-County Hospital had also started treating VL patients at this time, it was not included in this study because its VL treatment centre was not yet operational at the time of study design. The records of Kacheliba Sub-County Hospital listed the age, sex, village of residence, and month of VL diagnosis of all VL patients admitted for treatment, starting from January 1st, 2018. Admitted patients showed clinical signs of VL (such as chronic fever, splenomegaly and weight loss), and had been diagnosed with VL with an rK39 rapid diagnostic test (RDT), direct agglutination test and/or microscopic examination of splenic aspirate. The records did not specify which of these diagnostic tests had been used for each patient. In principle, all patients with a positive VL diagnosis are admitted for treatment according to the national guidelines for the management of VL [10]. Data from all VL patients admitted between January 1st, 2018 and December 9th, 2022 were manually copied into an Excel database. This was done by one researcher dictating the written data from the records, while a second researcher entered these data in Excel, followed by a cross-check to prevent errors. During data entry, patient data were anonymised by linking them to unique identifiers; patient names were not copied from the original records. The same two researchers were responsible for all data entry to ensure consistency.

## Patient characteristics

The Excel database of patient records was imported into the statistical software IBM SPSS Statistics version 28.0 (IBM Corp, Armonk, NY, USA) for data processing and analysis. For all VL patients recorded between 2018 and 2022, the sex ratio was calculated and age distribution was described using the median age and interquartile range (IQR). To explore a possible effect of age on the sex distribution of VL patients, sex ratio was also determined for different age groups: ≤1 year (infants), 2–5 years (young children), 6–10 years (school-aged children), 11–20 years (adolescents and young adults), 21–45 years (adults), and > 45 years (old adults). This structuration was chosen because of possible behavioural differences between males and females in these age groups, which could affect their risk of VL infection [18, 19].

## Temporal analysis

To analyse the temporal trend-cycle and potential seasonal pattern in VL admissions to Kacheliba Sub-County Hospital between 2018 and 2022, monthly VL admission numbers were plotted, and a seasonal decomposition analysis was performed in SPSS version 28.0. This procedure implements the ratio-to-moving-average method (Census Method I) and derives a seasonal, trend-cycle and residual component from the time series data of VL reports. This method was chosen for its simplicity, though its limitations include the assumption of a constant seasonal pattern over the study period, and over-smoothing of rapid rises and falls in the trend-cycle estimate [20]. A multiplicative model was applied to adjust for variation in the seasonal pattern amplitude proportional to the trend-cycle level. As VL admission numbers for December 2022 were only available until December 9th, the number of reports in these first 9 days were linearly extrapolated to 31 days.

### Spatial distribution of VL cases

The patients’ villages were georeferenced using a pre-established database from MSF, which listed the Global Positioning System (GPS) coordinates of the village of residence of VL cases from West Pokot and Amudat District (Uganda) between 2000 and 2010 [8]. For villages that were not listed in the MSF database, geographical coordinates were collected online from ICPAC Geoportal and Google Maps [21]. The georeferenced village locations and their annual number of VL cases were imported into ArcGIS Pro 3.2 software (ESRI, Redlands, CA, USA) and the total number of VL cases between 2018 and 2022 was mapped per village of residence.

To summarise the spatial distribution of VL in West Pokot at a higher administrative level and account for differences in population density, VL cases were aggregated per sub-location and the incidence was calculated using population counts from the 2019 Kenyan Housing and Population Census [15]. Sub-location incidences were mapped for the whole study period (per 1000 person-years) and for the separate years (per 1000 sub-location inhabitants).

### Village population estimation for spatial analysis

The spatial and spatiotemporal analyses were performed at the village level to identify VL hotspots and spatiotemporal clusters at the lowest possible geographical scale. This required calculating the VL incidence per village, in order to account for differences in village population. As village census data were unavailable, village population sizes were estimated using a WorldPop constrained population estimate grid for Kenya in 2020, at a resolution of 3 arc seconds (approximately 100 m, see Supplementary Fig. 1) [22]. To allocate a population estimate to the village point locations, Thiessen polygons were generated in West Pokot and neighbouring Kenyan counties with the Create Thiessen Polygons tool in ArcGIS Pro 3.2, based on all village locations that were listed in the ICPAC Geoportal dataset and the MSF database [8, 21]. Next, the sum of grid-cell population estimates within each Thiessen polygon was calculated to obtain a village population estimate (Supplementary Fig. 1).

Only georeferenced villages with at least one VL case between January 2018 and December 2022 were included in subsequent analyses of hotspots and spatiotemporal clusters. Several polygons were uninhabited based on the WorldPop data, and the corresponding villages were thus assigned a population of zero (Supplementary Fig. 1). None of these villages reported VL cases, therefore these false zeros did not affect the spatial and spatiotemporal analysis of village VL incidences.

### Hotspot analysis

To analyse the general spatial distribution pattern of VL incidence per year, village VL incidence was first tested for global spatial autocorrelation. This was done by calculating Global Moran’s Index (I) with the Spatial Autocorrelation tool in ArcGIS Pro 3.2. Global Moran’s I may range from − 1 to + 1, where − 1 signifies perfect geographical dispersion of high- and low-incidence VL villages, + 1 means perfect clustering of villages with similar VL incidence, and 0 means no spatial correlation. A *p* < 0.05 was considered statistically significant.

For the years in which global spatial autocorrelation of village VL incidence was detected, the Optimized Hot Spot Analysis Tool in ArcGIS Pro 3.2 was subsequently used to identify local spatial clusters of high-incidence villages (hotspots). This tool calculates the Getis-Ord Gi* statistic (z-score) by proportionally comparing the local sum of VL incidence in a village and its neighbouring villages with the sum of VL incidence over all VL-reporting villages. The p-value associated with the statistic’s z-score indicates the likelihood of the observed local VL incidence sum occurring under the null hypothesis that VL incidence is randomly distributed over all case-reporting villages. Hotspots were determined at the 90%, 95% and 99% confidence level.

### Spatiotemporal clustering analysis

Apart from spatial VL hotspots, temporal and local outbreaks may significantly contribute to ongoing disease burden and transmission as well. SaTScan scan statistics software v10.1 (Calverton, MD, USA), developed by Kulldorff et al. [23, 24], was used to retrospectively identify spatiotemporal clusters (outbreaks) of VL cases within West Pokot County and neighbouring Kenyan counties between January 2018 and November 2022; December 2022 was excluded from the analysis as the case data were truncated at December 9th. SaTScan simultaneously analyses the study region and period using a cylindrical scanning window, of which the circular base covers a geographic region in space and the height represents a time interval. The window moves through space and time while varying its base radius and height, thereby creating an infinite number of overlapping cylinders of different sizes and shapes within the study region and period. Each cylinder represents a potential cluster of villages in a certain time interval, with an associated aggregated VL incidence. Through Monte Carlo maximum likelihood testing, the likelihood of each space-time cluster is calculated under the assumption that all cases are randomly distributed in space and time over the total population of VL-reporting villages. This results in the identification of the most likely cluster rejecting this null hypothesis, represented by the smallest p-value, and potentially one or more secondary significant clusters.

In SaTScan, a discrete Poisson model for high rates was applied on monthly VL numbers of all VL-reporting villages with available GPS coordinates and population estimates, based on WorldPop data and village Thiessen polygons. The maximum spatial size of the window was set at 25 km, based on the estimated maximum daily travel distance of livestock-herding pastoralists in semi-arid areas of northern Kenya [25, 26]. Clusters were not allowed to overlap geographically. The maximum temporal cluster size was 50% of the study period (i.e., 2.5 years). The minimum number of cases per cluster was set at five; smaller clusters were considered irrelevant from a disease surveillance perspective. The Monte Carlo maximum likelihood testing was run for 9999 replications, and a significance level of *p* < 0.05 was used. Significant clusters were visualised using ArcGIS Pro 3.2.

## Results

### Patient characteristics

Between 2018 and 2022, 1948 VL patients were admitted to Kacheliba Sub-County Hospital. Patients were predominantly male (65.0%). Patient age ranged from 0 to 86 years and the median age was 10 years (IQR, 5–20 years). In patients younger than 6 years, the patient sex ratio was more similar than in older age groups (Table 1).

**Table 1:** Age distribution of VL patients at Kacheliba Sub-county Hospital between 2018 and 2022. Most VL patients were male, but this preponderance was less pronounced in infants and young children below the age of 6 years

Age group	Males (*n*)	% of all males	Females (*n*)	% of all females	Total	Male (%)^a^
≤ 1 year	27	2.1	28	4.1	55	49.1
2–5 years	274	21.6	206	30.2	480	57.1
6–10 years	317	25.0	137	20.1	454	69.8
11–20 years	347	27.4	142	20.9	489	71.0
21–45 years	256	20.2	142	20.9	398	64.3
> 45 years	45	3.6	25	3.7	70	64.3
Unknown	1	0.1	1	0.1	2	50.0
Total	1267	100.0	681	100.0	1948	65.0

^a^ Percentage of male patients per age group.

### Temporal patterns in VL admission numbers

**Fig. 2:**
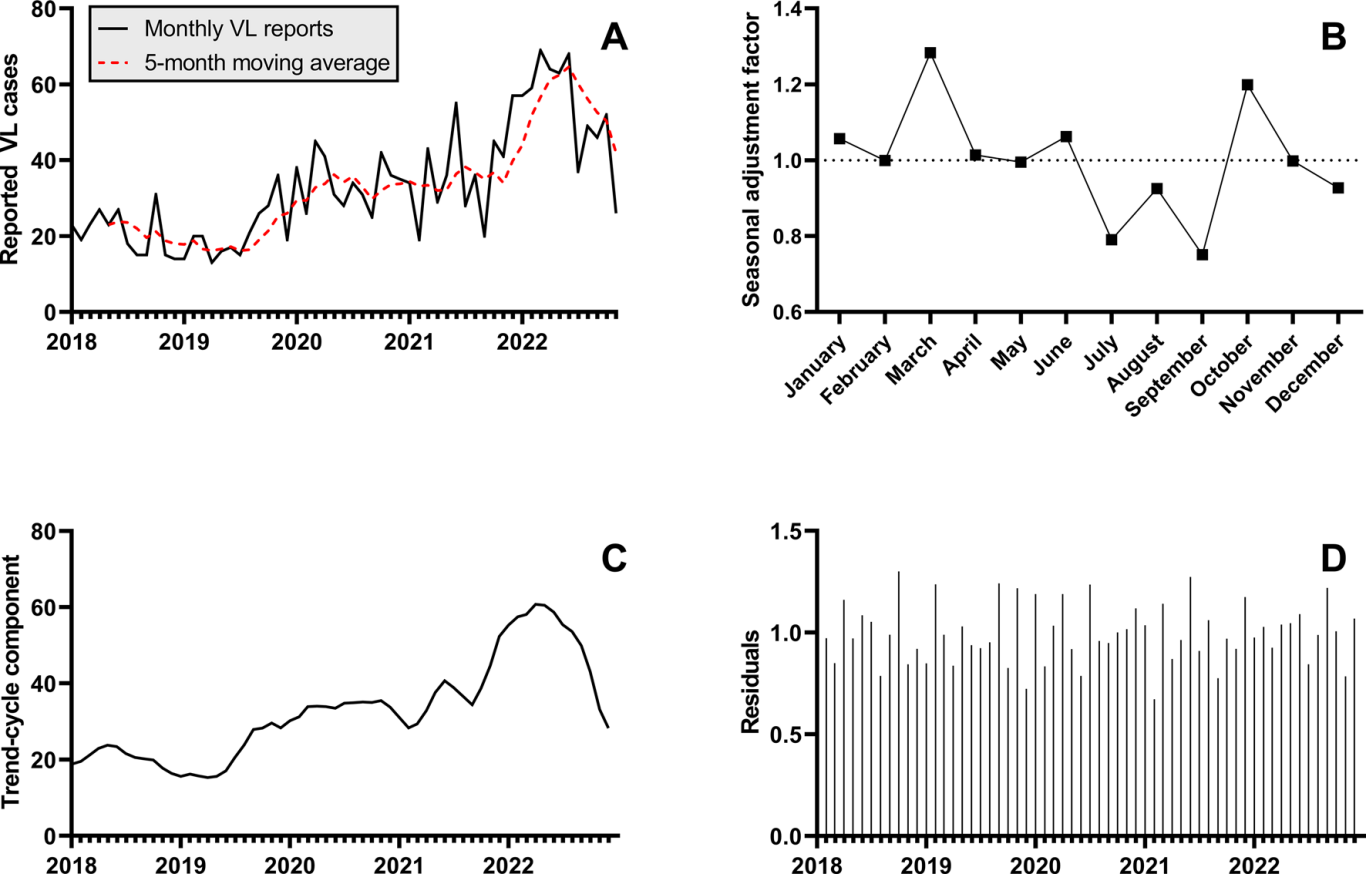
Seasonal decomposition of monthly VL admission numbers at Kacheliba Sub-County Hospital between 2018 and 2022. **(A)** Monthly VL reports at Kacheliba Sub-County Hospital and a 5-month moving average. **(B)** Seasonal component of the VL reports, expressed as relative adjustment factor. The graph shows how VL admissions tend to peak in March and October and drop between July and September, suggesting seasonality in VL transmission. **(C)** Trend-cycle component of the VL reports, which displays an increasing trend from mid-2019 until mid-2022 after adjustment for the seasonal and residual components. **(D)** Residual component after removal of trend-cycle and seasonal components. For the generation of the graphs in panels B – D, the incomplete VL reports for December 2022 (until December 9th, *n* = 8) were linearly extrapolated to 31 days (*n* = 28)

The annual number of VL admissions to Kacheliba Sub-County Hospital increased during the study period from 250 in 2018 to 598 in 2022 (Table 2; Fig. 2A). The multiplicative seasonal decomposition model demonstrated a seasonal pattern, with higher numbers in March and October and low numbers in July and September (Fig. 2B). These four months had a seasonal adjustment factor of 1.28, 1.20, 0.79 and 0.75, respectively. The trend-cycle component (Fig. 2C), adjusted for the seasonal pattern and residual random fluctuations (Fig. 2D), increased from May 2019 and reached its peak in April 2022, followed by a sharp decrease towards the end of 2022.

**Table 2:** Annual number of VL cases reported at Kacheliba Sub-county Hospital

Year	Reported cases	Georeferenced cases^a^	%
2018	250	188	75.2
2019	245	175	71.4
2020	412	295	71.6
2021	443	296	66.8
2022^b^	598	598	69.1
Total (2018 to 2022^b^)	1948	1367	70.2

^a^ Cases whose village of residence could be georeferenced with the available GPS databases.

^b^ Until December 9th

### Spatial distribution of VL cases

**Fig. 3:**
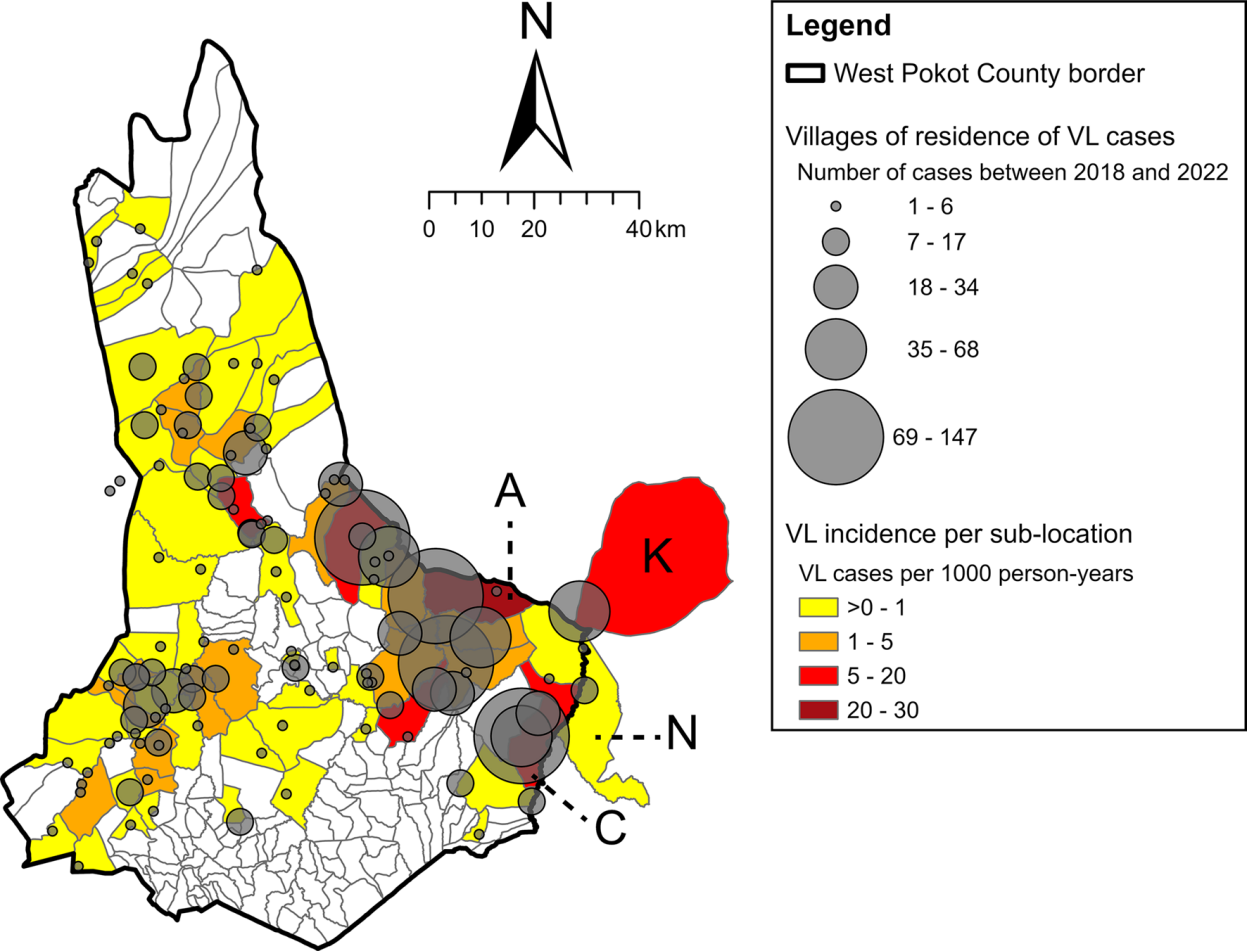
Number of VL cases reported between 2018 and 2022 per georeferenced village of residence. The sub-location colour represents the aggregated VL incidence per sub-location between 2018 and 2022. High-incidence sub-locations Amolem (A) and Chepserum (C) have been labelled, as well as Kakulit sub-location (K) in Turkana County and Ngaina sub-location (N) in Baringo County. Arror sub-location (Baringo County, three VL cases) lies outside of the map area. VL burden was highest in eastern regions, both in terms of absolute numbers as well as incidence per 1000 person-years

VL patients came from 513 different villages; for three patients, no village name was reported in the hospital records. All patients were reported to live in Kenya, except for two cases that came from Uganda; these were excluded from the spatial and spatiotemporal analyses. Using the available GPS data sources, 118 villages (23.0%) could be georeferenced. These represented 1367 (70.2%) of all 1948 VL patients reported between January 1st, 2018 and December 9th, 2022 (Table 2; Fig. 3). The demographics of the patients with village GPS coordinates were comparable to the complete patient population: median age was 10 years (IQR, 5–20 years) and 64.8% were male.

Among the 1365 georeferenced cases from Kenya, 1280 came from West Pokot County, 68 from Turkana County and 17 from Baringo County. They were distributed over 60 of the 224 sub-locations in West Pokot County, Kakulit sub-location in Turkana County, and Ngaina and Arror sub-locations (14 and 3 cases, respectively) in Baringo County (Fig. 3). Given the relatively high number of cases in Kakulit and Ngaina sub-locations and their adjacency to West Pokot, the VL cases from villages in these sub-locations were included in the analysis of hotspots and spatiotemporal clusters. Amolem sub-location in the east of West Pokot had the highest VL incidence, with 30.1 cases per 1000 person-years (Fig. 3). Other high-incidence sub-locations included Chepserum in the southeast (18.9 cases per 1000 person-years) and Kakulit (15.7 cases per 1000 person-years) in Turkana County. No VL cases were observed in the central southern regions of West Pokot.

**Fig. 4:**
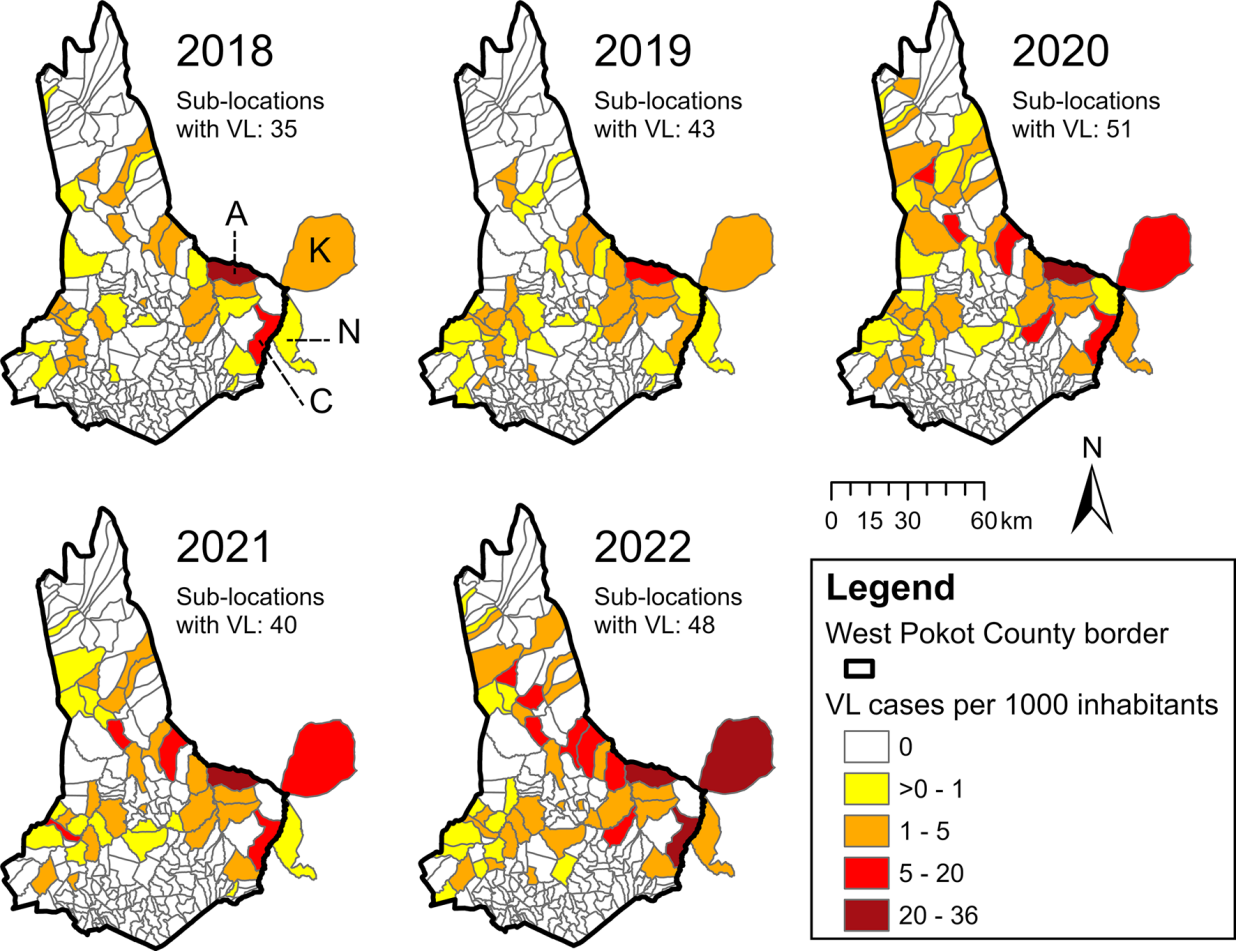
Annual VL incidence by sub-location in West Pokot County and neighbouring Kakulit (K) and Ngaina (N) sub-locations. High-incidence sub-locations Amolem (A) and Chepserum (C) have been labelled. Arror sub-location (Baringo County, three VL cases) lies outside of the map area, and two cases from Uganda are not shown. In all study years, the highest VL incidence was found in eastern sub-locations, of which several showed an increasing incidence trend over time

Mapping the VL incidence per year at sub-location level revealed that the georeferenced VL cases were spatially most dispersed in 2020, across 49 sub-locations in West Pokot County as well as Kakulit and Ngaina sub-locations (Fig. 4). From 2020 to 2022, several sub-locations in the east and north of West Pokot showed an increase in VL incidence.

### Global spatial autocorrelation and VL hotspots at village level

Global spatial autocorrelation of VL incidence per 1000 village inhabitants was detected in all study years (Table 3). The positive Moran’s I values with significant z-scores indicated a clustered pattern of VL incidence rates over the patient villages between 2018 and 2022. Clustering was most prominent in 2019 (Moran’s I = 0.115385).

**Table 3:** Assessment of spatial autocorrelation of VL incidence in case-reporting villages using global Moran’s I. A clustered pattern was detected for all study years

Year	Moran’s I	Expected I	Variance	Z-score	*P*-value	Pattern
2018	0.103463	-0.008850	0.000643	4.429790	0.000009	Clustered
2019	0.115385	-0.008850	0.000637	4.921081	0.000001	Clustered
2020	0.079723	-0.008850	0.000611	3.583781	0.000339	Clustered
2021	0.090537	-0.008850	0.000553	4.227929	0.000024	Clustered
2022	0.073275	-0.008850	0.000451	3.868042	0.000110	Clustered

**Fig. 5:**
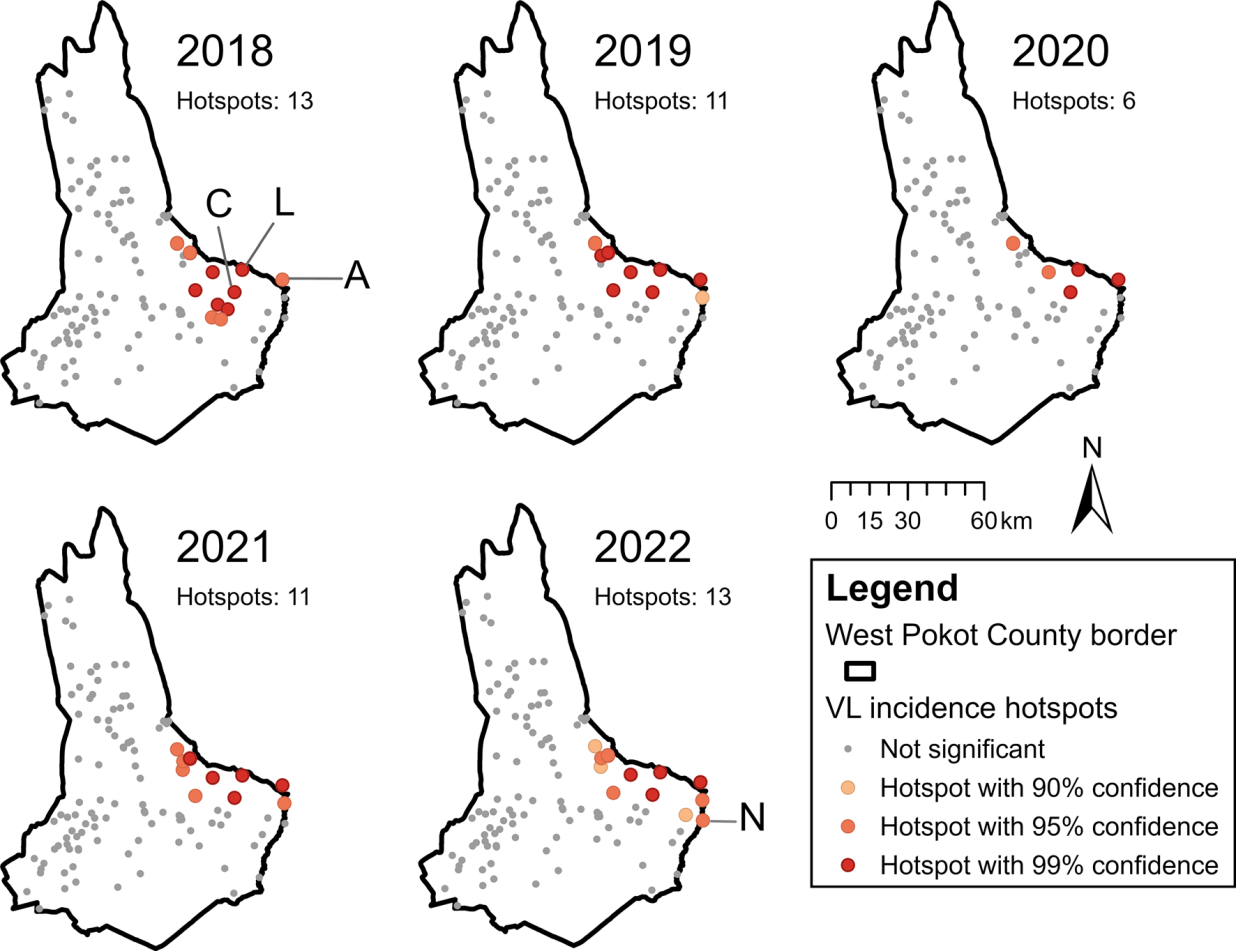
Hotspot villages of VL incidence from 2018 to 2022 identified using Getis-Ord Gi* analysis. The villages Akulo (A), Chepaywat (C), Lotongot (L) and Ngaina (N) have been labelled. In all study years, VL hotspots were found in eastern villages

As all years displayed global spatial autocorrelation of VL incidence, Getis-Ord Gi* analysis was performed for each year to describe the underlying local clustering pattern at the village level. This resulted in the identification of 16 hotspot villages in the east of West Pokot between 2018 and 2022, as well as Akulo village (in all years) and Ngaina village (in 2022) directly across the county border with Turkana County and Baringo County, respectively (Fig. 5). The villages Lotongot and Chepaywat were marked as hotspots at a 99% confidence level in all study years. The number of hotspots decreased between 2018 and 2020 but increased again in 2021 and 2022 (Fig. 5). However, their spatial distribution remained largely consistent over time.

### Spatiotemporal clustering analysis

**Fig. 6:**
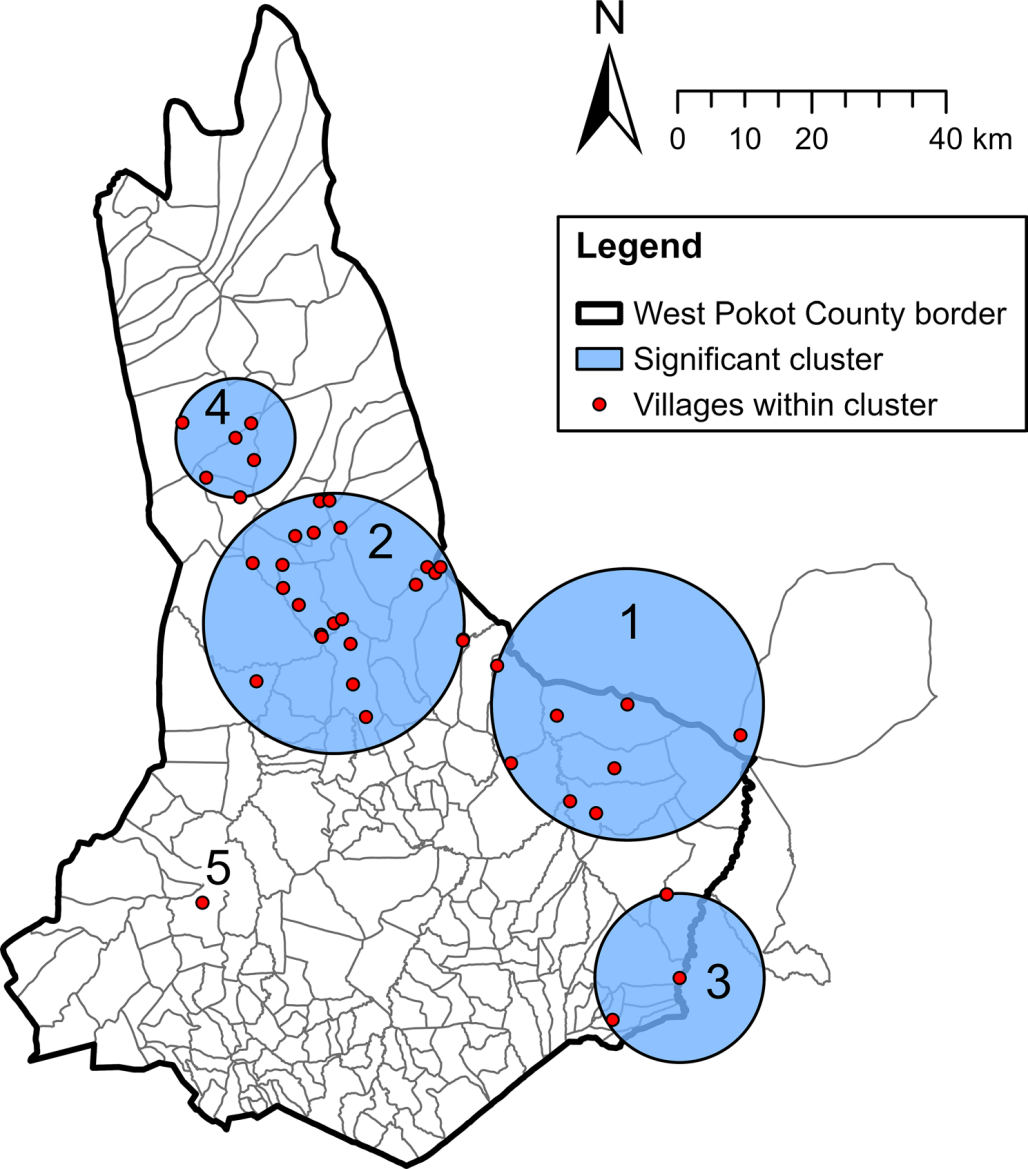
Spatiotemporal clusters of villages with high VL incidence between January 2018 and November 2022. Clusters were identified with SaTScan discrete Poisson model; their numbering corresponds with the numbers in Table 4. The spatiotemporal clusters can be considered as local VL outbreaks. The location of clusters 1, 2 and 3 overlaps with many of the high-incidence sub-locations as shown in Fig. 4.

With the exclusion of two villages in Uganda and Arror village in Baringo County, the SaTScan discrete Poisson model was run on 115 villages with one or more VL cases between January 2018 and November 2022. The model identified one most likely spatiotemporal cluster (cluster 1) and four secondary clusters (clusters 2 to 5, Table 4; Fig. 6). The clusters can be considered as local VL outbreaks. Cluster 1 occurred in the east of West Pokot between June 2020 and October 2022, with a radius of 20.23 km. It included 287 observed cases, where 9.69 cases were expected (relative risk: 37.33, *p* < 0.001). The age and sex distribution of the most likely cluster was similar to the total VL patient population (Table 4). This cluster represented 23.2% of all cases reported between June 2020 and October 2022 (*n* = 1238) and largely overlapped with the hotspot villages of these years (compare Figs. 5 and 6). The secondary clusters were located in the centre, southeast, north and west of West Pokot County (Table 4; Fig. 6). Cluster 3 consisted of relatively young VL patients. The only cluster before 2020 was cluster 5, which was situated in a single village.

**Table 4:** **Spatiotemporal clusters of VL cases identified with SaTScan discrete Poisson model.** Five statistically significant spatiotemporal clusters (outbreaks) were identified. The cluster numbering corresponds with the numbers in Fig. 6.

Cluster	Radius (km)	Time frame	Total cluster population^a^	Cases	Male (%)	Median age (years) [IQR]	% of total^b^	Expected cases	RR	*p*-value
1 (MLC)	20.23	6/2020 to 10/2022	2,403	287	61.7	10 [5–20]	23.2	9.69	37.33	< 0.001
2	19.39	10/2020 to 11/2022	13,783	194	70.5	10 [5–15]	16.9	49.76	4.38	< 0.001
3	12.59	2/2022 to 6/2022	15,733	39	56.4	6 [3–12]	12.1	10.77	3.70	< 0.001
4	8.86	2/2020 to 9/2020	3,574	21	61.9	8 [5–16]	8.0	3.96	5.37	< 0.001
5	NA^c^	1/2018 to 3/2020	282	10	60.0	15 [7–19]	1.7	1.06	9.53	0.019

MLC: most likely cluster; NA: not applicable; RR: relative risk

^a^ Population of villages in cluster that reported at least one VL case between 2018 and 2022

^b^ Proportion of all VL cases reported in the cluster period

^c^ Cluster consisted of a single village

## Discussion

This study has provided novel insights into the basic demographics and spatiotemporal patterns of VL infections in West Pokot County, the major endemic focus of this disease in Kenya. Between 2019 and 2022, there was a considerable increase in VL reports in West Pokot and neighbouring regions. High-incidence villages, clustered in the east of West Pokot, and several local outbreaks since 2020 appear responsible for the rise in VL admissions at Kacheliba Sub-County Hospital.

The sex ratio and age distribution of VL cases in West Pokot were similar to what has previously been reported for this region [8, 27]. The preponderance of male VL patients may have multiple causes. Boys and men are often responsible for livestock herding, and thereby more likely to be exposed to infectious sandfly bites by disturbing their resting sites outside domestic areas [19]. Moreover, some studies have suggested that males are biologically more susceptible to VL, potentially through the effect of sex hormones [28–32]. Remarkably, this study found more balanced sex ratios in the youngest age groups. This could support the hypothesis that the sex disparity in VL infections results from behavioural and hormonal differences, as these are likely to be less prominent at younger ages. In addition, female patients of adolescent age and older may also be underrepresented in the hospital records: they frequently have less access to healthcare than males and show more delay in healthcare seeking, due to their subordinate position in Pokot culture [33].

The number of VL admissions at Kacheliba Sub-County Hospital increased from mid-2019 and peaked in the first half of 2022. Thereafter, monthly numbers started to decline, although this decrease may have been biased by the opening of a second VL treatment centre in Sigor Sub-County Hospital in September 2022. The overall trend in VL admissions remained visible after adjustment for seasonality and random variations, and corroborated the VL outbreak reported in West Pokot between 2020 and 2022 by the MoH [5]. In this period, there were 1453 VL cases registered in the Kacheliba Hospital records, which is significantly more than the 930 cases officially reported by the MoH [5]. This suggests that a considerable amount of cases was missed by the country’s reporting system, which may require re-evaluation and optimisation. To improve reporting of case numbers by VL treatment facilities, staff will need to be appointed and trained for this specific task. In addition, the national control programme currently manages VL reporting data through multiple systems, including KHIS, the WHO integrated data platform, and county situational reports [4]. The resulting data inconsistencies may be prevented by the design and implementation of a tailored, centralised VL data management system.

Multiple factors may have played a role in the increase in VL case numbers. In general, VL incidence in East Africa tends to have a cyclic pattern, with a rise in cases during two to five years followed by a decrease [34]. These repeating fluctuations are believed to result from migration of naïve or infected populations, which promotes increased VL transmission that will eventually diminish due to acquired immunity in the population [35–37]. Human migration may also have sparked the outbreak in West Pokot described herein, although there are no specific data available to support this hypothesis. However, temperatures and rainfall in West Pokot have become increasingly variable in recent years, and these climate changes are likely to affect livestock herder migration as well as sandfly populations [38]. Future studies should investigate potential associations between VL incidence and climatic and environmental factors in West Pokot. Their outcomes could elucidate causes of past outbreaks, and thereby support epidemic preparedness in the future.

Seasonal decomposition analysis revealed that VL admission numbers in March and October were generally more than 20% above the seasonally-adjusted trend-cycle. These peaks coincide with the end of the dry seasons and the beginning of the long rains (March) and short rains (October). Since the sandfly vector of *L. donovani* in Kenya, *P. martini*, is believed to be most abundant during the rainy seasons, VL transmission is potentially higher in these periods [39, 40]. However, there may be a lag in increasing VL hospital admissions due to the long incubation period of the disease, varying from 2 to 6 months. The peak in March could, therefore, reflect increased transmission during the short rains (October – December), while the October peak may result from higher transmission during the long rains (March – June). The seasonal pattern in VL hospital admissions observed here was not seen in previous data from 2000 to 2010 as reported by Mueller et al., although this earlier study did not perform seasonal decomposition analysis [8]. More research is therefore needed to confirm the seasonality hypothesis, for instance through longitudinal sandfly trapping and screening of their *L. donovani* infection rates over the year.

VL incidence mapping and hotspot analysis demonstrated significant spatial clustering of VL cases in the eastern regions of West Pokot. The most likely spatiotemporal cluster of VL cases was also located in this area. This cluster constituted a local VL outbreak of 2 years and 5 months, during which more than 10% of the estimated population of VL-reporting villages in this cluster was infected with VL. These cases represented nearly a quarter of all VL admissions at Kacheliba Sub-County Hospital in this period. This most likely cluster, together with secondary clusters 2 and 3, significantly contributed to the overall increase in VL reports between 2020 and 2022. The VL focus in the east of West Pokot is a remarkable contrast with the period 2000 to 2010, when most VL patients were reported in the west of the county [8]. There may be multiple causes of this apparent spatial shift. VL awareness campaigns, organised by the national leishmaniasis control programme in communities around Kacheliba, may have led to destruction of sandfly breeding sites (like termite mounds) and improved healthcare seeking behaviour, which can both contribute to reduced disease transmission [4]. Moreover, high disease exposure in the past may have led to widespread VL immunity in western areas of West Pokot. The reasons behind the high VL transmission in the east will require further research. Possible predisposing factors may include high poverty rates, limited knowledge of VL among local communities, and favourable ecological conditions for the sandfly vector. During the studied period, insufficient access to VL care may also have played a role, as Sigor Hospital only started providing this in late 2022.

The rising VL numbers in the study period underpin how VL control in West Pokot must be intensified, particularly in the hotspot regions in the east of the county. High-incidence villages, such as Lotongot and Chepaywat, should be prioritised for control interventions, including community sensitisation campaigns and evidence-based vector control measures. In close collaboration with Turkana County and Baringo County, these interventions should also be implemented in the hotspot villages Akulo and Ngaina. Furthermore, access to VL care in the east of West Pokot could be further improved, for instance by decentralisation of diagnostic testing with rK39 RDTs. The demonstration of seasonality in VL hospital admissions could help to better allocate (human) resources and manage the supply chain of therapeutics and diagnostic tests to treatment centres. Finally, regular mapping of VL reports from both Kacheliba and Sigor hospital could be a feasible first step to improve VL surveillance as part of the Kenyan national leishmaniasis control programme.

Collection of complete and accurate location data in the field is challenging in remote regions such as West Pokot due to poor infrastructure and limited resources. Therefore, this study made use of existing databases on village locations, through which villages of residence could be georeferenced for 70.2% of the VL cases admitted to Kacheliba Sub-County Hospital between 2018 and 2022. This subsample was considered representative for the overall patient population, as it had a similar age and sex distribution. However, 77.0% of the recorded villages could not be georeferenced. Although the missed villages represented relatively few VL patients (29.8%) and may thus have been small, the incomplete georeferencing may have biased some of the study results. This shortcoming followed from the use of handwritten hospital records as data source, which came with challenges such as legibility issues, multiple spellings of the same village name, and alternative local village names. To improve georeferencing of VL case villages in the future, a predefined list of standardised village and sub-location names should be used to record the residence of VL patients at treatment centres. If this is done digitally, the data would be easily accessible for surveillance purposes as well.

This study used village population estimates, based on WorldPop data and Thiessen polygons, for the calculation of VL incidence at the village level. Inaccuracies in these estimates may have affected the incidence rates and subsequently the spatiotemporal analysis outcomes, especially in sparsely populated regions with high numbers of VL cases. Nevertheless, it was considered a feasible and reliable approach to correct VL numbers according to village population size. Another limitation of this study was the collection of patient data from the records of a single hospital, meaning that patients from more distant regions may have been underrepresented in the dataset. Due to this possible spatial bias, potential hotspots and outbreaks in remote areas, such as the north of West Pokot, may have been missed. Lastly, patient characteristics apart from age and sex, such as occupation and socio-economic status, were not available, limiting a more elaborate description of hotspots and spatiotemporal clusters.

## Conclusion

This study has demonstrated that VL remains highly prevalent in West Pokot and continues to affect vulnerable populations, such as children and adolescents. High incidence and local outbreaks in eastern sub-locations call for targeting these hotspot regions for intensified control measures. These interventions should be community-based, and implemented through involvement of local community leaders. The successful use of hospital record data for spatiotemporal mapping of VL could serve as an example for the leishmaniasis control programme in Kenya, as well as other endemic countries in East Africa. Finally, future research may capitalise on this study’s findings by investigating the underlying determinants of high VL transmission in hotspot areas, which could inform the development of outbreak prediction models.

## Electronic supplementary material

Below is the link to the electronic supplementary material.

Supplementary Material 1

## Data Availability

The datasets used and/or analysed during the current study are available from the corresponding author on reasonable request.
