# Supplementary material for: An epidemiological and spatiotemporal analysis of visceral leishmaniasis in West Pokot, Kenya, between 2018 and 2022
Source: BMC Infect Dis. 2024 Oct 16;24:1169. doi: 10.1186/s12879-024-10053-4 (PMC11484382; doi:10.1186/s12879-024-10053-4)
Supplement: Supplementary file 1 — Supplementary Material 1 [file 12879_2024_10053_MOESM1_ESM.docx]

Supplementary material


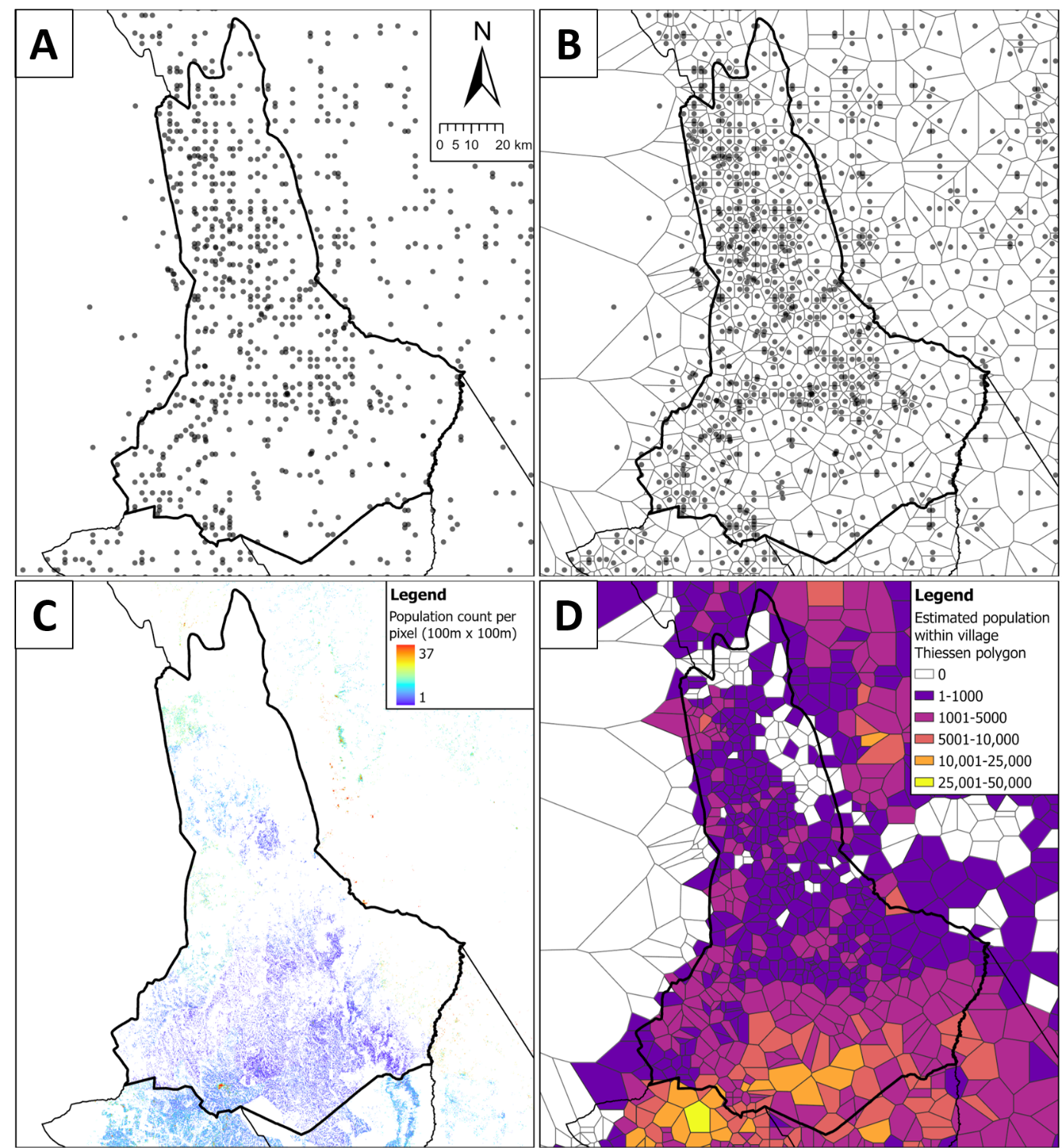


**Supplementary Figure 1: Generation of village population estimates using Thiessen polygons and WorldPop data.** (A) Locations of all villages reported in the ICPAC Geoportal dataset of village locations in Kenya and the MSF database with villages of residence of VL patients, reported between 2000 and 2010 (Mueller et al., 2014); (B) Thiessen polygons generated based on the village locations; (C) WorldPop constrained population estimate grid for the study area (2020), at a resolution of 3 arc second (approximately 100 m); (D) aggregated WorldPop population per village Thiessen polygon, which was used to calculate VL incidence per village.
